# A Quantitative MRI Method for Imaging Blood-Brain Barrier Leakage in Experimental Traumatic Brain Injury

**DOI:** 10.1371/journal.pone.0114173

**Published:** 2014-12-05

**Authors:** Wei Li, Justin Alexander Long, Lora Talley Watts, Zhao Jiang, Qiang Shen, Yunxia Li, Timothy Q. Duong

**Affiliations:** 1 Research Imaging Institute, University of Texas Health Science Center at San Antonio, San Antonio, Texas, United States of America; 2 Department of Ophthalmology, University of Texas Health Science Center at San Antonio, San Antonio, Texas, United States of America; 3 Department of Cellular and Structure Biology, University of Texas Health Science Center at San Antonio, San Antonio, Texas, United States of America; 4 Department of Neurology, University of Texas Health Science Center at San Antonio, San Antonio, Texas, United States of America; 5 South Texas Veterans Health Care System, Department of Veterans Affairs, San Antonio, Texas, United States of America; University of Michigan, United States of America

## Abstract

Blood-brain barrier (BBB) disruption is common following traumatic brain injury (TBI). Dynamic contrast enhanced (DCE) MRI can longitudinally measure the transport coefficient K^trans^ which reflects BBB permeability. K^trans^ measurements however are not widely used in TBI research because it is generally considered to be noisy and possesses low spatial resolution. We improved spatiotemporal resolution and signal sensitivity of K^trans^ MRI in rats by using a high-sensitivity surface transceiver coil. To overcome the signal drop off profile of the surface coil, a pre-scan module was used to map the flip angle (B_1_ field) and magnetization (M_0_) distributions. A series of T_1_-weighted gradient echo images were acquired and fitted to the extended Kety model with reversible or irreversible leakage, and the best model was selected using F-statistics. We applied this method to study the rat brain one hour following controlled cortical impact (mild to moderate TBI), and observed clear depiction of the BBB damage around the impact regions, which matched that outlined by Evans Blue extravasation. Unlike the relatively uniform T_2_ contrast showing cerebral edema, K^trans^ shows a pronounced heterogeneous spatial profile in and around the impact regions, displaying a nonlinear relationship with T_2_. This improved K^trans^ MRI method is also compatible with the use of high-sensitivity surface coil and the high-contrast two-coil arterial spin-labeling method for cerebral blood flow measurement, enabling more comprehensive investigation of the pathophysiology in TBI.

## Introduction

The blood-brain barrier (BBB) plays a vital role in regulating the entry of blood-borne factors and circulating immune cells into the brain, hereby providing a highly stable biochemical environment for the normal functioning of neuronal cells. The integrity of the BBB can be disrupted by the translational and rotational forces in traumatic brain injury (TBI). The resulting BBB damage can evolve dynamically in both time and space with studies showing multi-phasic characteristics [1]. BBB leakage can lead to an imbalance of electrolytes, edema formation, inflammation, etc., and ultimately delayed neuronal dysfunction and degeneration [2]. There is a growing consensus that post-traumatic BBB disruption is one of the major factors that contribute to increased severity of TBI [3]. BBB has also been suggested as a target for therapeutic invention, as a normally functioning BBB is important for restoring brain hemostasis, and provides an optimal environment for neuronal repair [2], [4].

Quantitative assessment of BBB permeability is of particular importance for studying the disease pathophysiology and for optimizing therapeutic interventions in TBI. Traditional histological Evans Blue extravasation has been widely used to measure BBB leakage following TBI [1], [5], [6]. However, this method requires the sacrifice of the animals and does not permit for longitudinal assessments. Dynamic contrast enhanced (DCE)-MRI provides a promising alternative for noninvasive longitudinal assessment of the opening of BBB [7]. Unlike conventional T_1_-weighted images or T_1_ maps that are influenced by multiple non-physiological factors, e.g. timing, image acquisition, etc., DCE-MRI provides quantitative assessment of the diffusive transport of T_1_-enhancing agents, e.g. gadolinium-diethylenetriamine pentaacetic acid (Gd-DTPA), across the BBB. By fitting the mechanistic tracer kinetic models, DCE-MRI yields the volume transfer coefficient K^trans^, the extracellular extravascular space *v*_e_, etc. Currently, there is a consensus that K^trans^ best reflects tissue permeability alterations and should be the primary end point of data fitting of DCE-MRI [7], [8].

K^trans^-MRI has been widely used to study stroke [9], [10], [11] and, to a much lesser extent, TBI. A recent study reported that K^trans^ correlated with T_2_ lesion volume and the extent of injury in a rabbit weight-drop model of TBI [12]. A subsequent longitudinal study revealed different trajectories of BBB evolutions in focal and perifocal lesion areas using the same TBI model [13]. The focal lesion area showed increased K^trans^ 3 h post-TBI, that peaked at 3 days, and remained higher than sham-operated animals at 7 and 30 days, while the perifocal lesion area showed increased K^trans^ at 1, 3 and 7 days, and normalized K^trans^ at 30 days [13]. Although there is a need for systematic investigation of post-traumatic BBB evolution under various conditions, K^trans^ MRI is still not widely used in experimental or clinical TBI, because it is generally noisy and has low spatial and temporal resolution.

In this study, we improved the K^trans^ MRI method by improving signal-to-noise ratio (SNR) and spatiotemporal resolution for longitudinal evaluation of BBB alterations in experimental TBI. To achieve the desired SNR, we used a high-sensitivity surface transceiver coil and a high field Bruker 7T scanner. To overcome the signal drop off profile associated with the use of a surface coil, the flip angle and the M_0_ distributions were measured using an optimized pre-scan module. The dynamic data were acquired using a fast gradient echo sequence. The arterial input function (AIF) was determined using the data and the scaling approach described in [14], [15]. The K^trans^ map was derived by fitting the data to the extended Kety model with irreversible or reversible leakage, followed by model selection using F-statistics. We applied this method to an established controlled cortical impact model of TBI, and compared spatial profiles of BBB permeability changes against Evans Blue extravasation and cerebral edema indicated by T_2_ abnormality.

## Materials and Methods

### Animal Models

All animal procedures were approved by the Institutional Animal Care and Use Committee of University of Texas Health Science Center at San Antonio. TBI was performed using a controlled cortical impact (CCI) model as described previously [16], [17]. Briefly, male Sprague Dawley rats (250–350 g, n = 3) were anesthetized initially with 5% isoflurane mixed with room air and maintained at 1.5% isoflurane throughout all surgical procedures. A Ø5 mm craniotomy was produced over the left forelimb somatosensory cortex (S1: 0.25 mm anterior and 3.5 mm lateral to bregma), revealing the dura mater. The intact dura mater was impacted (Precision Systems and Instrumentation, LLC, Fairfax Station, Virginia) with a Ø3 mm tip (5.0 m/s, 250 µs dwell time, 1 mm depth) to emulate a mild focal TBI. The cranial opening was sealed with bone wax following the impact.

### MRI

MRI was performed on a high field Bruker 7 Tesla BioSpec MRI scanner with a custom-made surface coil for brain imaging. The animal was secured in a custom built, MRI compatible, rat head stereotaxic holder with ear and tooth bars, and was scanned under 1.2% isoflurane. End-tidal CO_2_ was monitored via a SurgiVet capnometer (Smith Medical, Waukesha, WI, USA). Noninvasive end-tidal CO_2_ values have been calibrated previously against invasive blood gas samplings under identical settings. The heart rate and blood-oxygen saturation level were also monitored using a MouseOx system (STARR Life Science, Oakmont, PA, USA).

#### K^trans^-MRI (19.4 mins)

All K^trans^ MRI data were acquired using a 2D multi-slice FLASH sequence. A pre-scan module was used to determine the flip angle and M_0_ distribution, which includes 3 FLASH scans with different TRs: 64 ms (scan 1), 200 ms (scan 2) and 3000 ms (scan 3). The rest of imaging parameters are: five 1.0-mm coronal slices, TE = 2 ms, FOV = 2.2×2.2 cm^2^, 128×128 data matrix and 30^o^ nominal flip angle. The pre-scan takes 7.4 min. Dynamic scans use a TR of 64 ms, and otherwise identical sequence parameters. After baseline data were acquired for 2 min, a bolus (0.2 ml/kg) of gadodiamide (GE Healthcare, USA) was injected intravenously through the tail vein, during which the dynamic scan was continued. A total of 90 dynamic images were acquired with a temporal resolution of 8 s, lasting 12 min total.

#### T_2_ map (9.5 mins)

T_2_-weighted images were acquired using fast spin-echo (FSE) sequence with TR = 3 s (90^o^ flip angle), effective TE = 18, 54, 90 and 126 ms, 4 echo train length. The other parameters were: seven 1.0-mm coronal images, FOV = 2.56×2.56 cm^2^, matrix 96×96 and reconstructed to 128×128, and 8 transients for signal averaging.

### K^trans^ Mapping

The steady-state spoiled gradient echo (GRE, acquired using the 2D FLASH sequence) signal amplitude for a given TR (M_TR_) can be related to M_0_, R_1_ and α as follows:
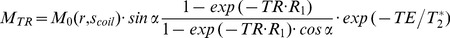
(1)where 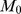 is a function of both spatial location *r* and coil sensitivity 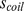. To minimize the R_2_^*^ signal decay, a single-echo GRE scan was used with minimum TE and a low dose of gadolinium. As an approximation, 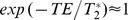 was used in the subsequent calculation. A pre-scan module composed of three GRE scans was used to determine flip angle (α) and tissue magnetization (M_0_).

**Figure 1:**
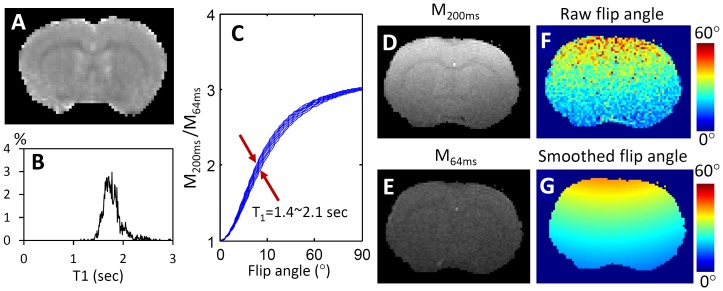
Determination of flip angle distribution. (A) T_1_ map of a healthy rat brain acquired using an inversion recovery sequence. (B). Histogram of T_1_ distribution in the brain, showing the majority of T_1_ was within the range of 1.5 to 2.1 s. (C) The relationship between the ratio of M_200 ms/_M_64 ms_, T_1_ and flip angle. This simulation showed that the ratio depends weakly on T_1_ by mainly on the flip angle distribution. (D) and (E) were the magnitude of GRE signal at TR of 200 and 64 ms. (F) The raw flip angle map determined from the ratio of M_200 ms/_M_64 ms_. (G) The final flip angle map after a smooth fitting.

For flip angle mapping, simulations using Eq. 1 show that the ratio of GRE magnitudes at TRs of 200 ms and 64 ms depends strongly on α, but very weakly on T_1_ (Fig. 1C). Given the narrow T_1_ distribution of brain tissue, a raw α (or B_1_^+^) map can be obtained from the ratio map with a fixed T_1_ value. Assuming α is a smooth function of location, the final α map can be obtained after smoothing.

For M_0_ determination, according to Eq. 1, the GRE signal becomes less dependent on T_1_ with increasing TR. With a long TR of 3000 ms, GRE magnitude depends primarily on sin(*α*) for small to medium flip angles (α<45°), but very weakly on T_1_. Given the normal flip angle of 30°, the actual flip angle of the brain tissue is within the range of 15 to 45°; therefore, M_0_ can be determined from the long TR scan and the flip angle distribution as follows: 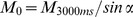
(2)


Given the smooth α distribution, high-SNR M_0_ map, and dynamic scans using sensitive surface coil at high field, dynamic R_1_ map was obtained using Eq. [1] with sufficient SNR.

The AIF was determined using the data and the scaling approach by Ewing and colleagues [14], [15]. Briefly, the mean AIF (*AIF*_mean_) was measured in a group of male rats (approximately 300 g) using the custom-synthesized radiolabeled Gd-DTPA [14]. Assuming the plasma volume was 1% and there was no BBB leakage in the contralesional side of caudate-putamen (CPU), the AIF was determined using the following scaling: 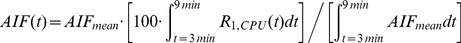
(3)


The R_1_ and the AIF were then used to fit the extended Kety model: 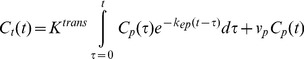
(4)where 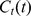 and 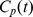 are tissue and plasma gadolinium concentrations, and 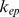 is the reversible mass transfer coefficient. In this study, the R_1_ and scaled AIF values were directly used for data fitting without further conversion to concentrations using the relativity of contrast agent. Since it is difficult to obtain acceptable K^trans^ maps by simultaneously fitting all three parameters, we used the model selection approach by Ewing and colleagues [15] to select a simpler model that could sufficiently describe the dynamic contrast change. Data was fit to the extended Kety model with the two following assumptions as described by Ewing and colleagues [15]: 
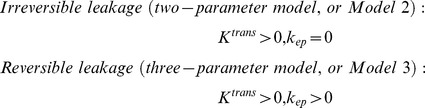
(5)


For model selection, the irreversible model is selected, unless the reversible model yields a statistically significant better fit. The cut-off criteria use the F-statistic, which can be calculated using the summed squared residues (SSE) and the number of samples (N) is as follows:
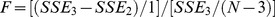
(6)where the subscript represents the corresponding model. The final selection mask is determined as:
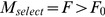
(7)


The threshold for F-statistic (F_0_) was set as 10 (P<0.05). The final K^trans^ map was then determined as: 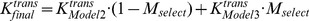
(8)


All the calculations were performed using Matlab R2011b (Mathworks, Natick, MA).

### Histology

BBB permeability was evaluated using Evans Blue (EB) extravasation as described previously [18], [19]. Briefly, 1 mL of 4% EB (Sigma-Aldrich, USA) solution was injected into the tail vein immediately after the dynamic scan and allowed to circulate for 1 hour. The rats were then transcardially perfused with heparinized saline solution followed by 4% paraformaldehyde. Brains were removed and cut into 1 mm slices to match the slice orientation and position of the MRI scans. The brain sections were imaged using an optical scanner. The brain regions stained with blue color indicate the leakage of EB bound to plasma albumin that moved across the impaired BBB.

### Statistics

Data was presented as the mean ± standard error of the mean. Unpaired Student t-tests were used for comparisons between K^trans^ values determined by different methods. Bonferroni correction was used to correct P-values for multiple comparisons. To evaluate the nonlinearity of K^trans^-T_2_ relationship, the data was fit to a quadratic function, using the Matlab statistics toolbox, which gives both the coefficient and the corresponding P-value. Statistical significance was set at P<0.05.

## Results

### Flip angle and M_0_ determination

Fig. 1A shows a T_1_ map of a rat brain obtained using an inversion recovery method. The T_1_ values were in the range of 1.5 to 2.1 s (using a threshold of 0.3% according to the histogram shown in Fig. 1B), with a mean of 1.8 s. Given this narrow range of T_1_ variation, the ratio of *M*_200 ms_/*M*_64 ms_ was dominated by the flip angle variation and was not significantly affected by the T_1_ distribution (Fig. 1C). Assuming a fixed T_1_ value of 1.8 s, a raw flip angle map (Fig. 1F) was determined from the ratio of *M*_200 ms_ (Fig 1D) and *M*_64 ms_ (Fig. 1E). The final flip angle distribution was then obtained after a smooth fitting (Fig. 1G).

**Figure 2:**
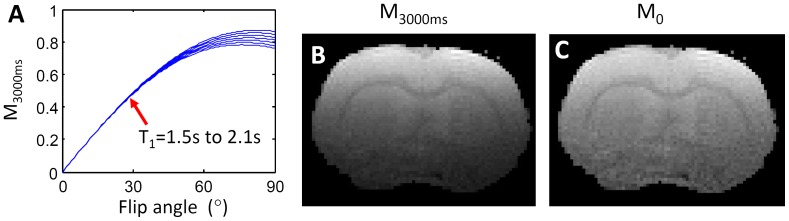
Quantification of M_0_. (A) Simulation showed that at long TR of 3000 ms, the GRE signal was primarily dependent on flip angle for small to medium flip angles (<45^o^), but with negligible dependence on the T_1_. (B) Magnitude of GRE signal at TR of 3000 ms. (C) M_0_ map derived by dividing M_3000 ms_ by sin(α).

As shown in Fig. 2A, at a long TR of 3000 ms, the *M*_3000 ms_ was primarily determined by the flip angle, but was not on significantly affected by the T_1_ distribution. Therefore, the M_0_ distribution (Fig. 2C) was determined by dividing the *M*_3000 ms_ (Fig. 2B) by the sine of the flip angle (Fig. 1G).

### Dynamic R_1_ mapping

**Figure 3:**
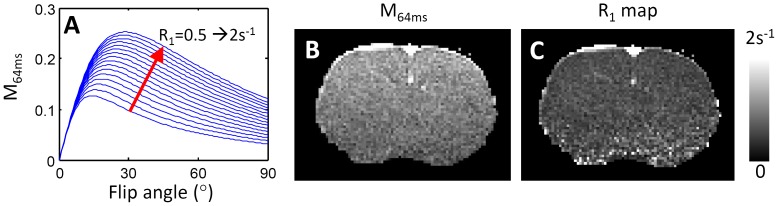
R_1_ mapping. (A) Dependence of GRE signals on R_1_ and flip angles. (B) T_1_-weighted image. (C) R_1_ map.

Fig. 3A simulated the changes of GRE signal with the flip angle and R_1_ values using Eq. 1. The GRE signal showed good sensitivity to R_1_ changes for flip angles larger than 15^o^, which can be satisfied by the majority of brain tissue as shown in Fig. 1G. Given flip angle and *M*_0_ distribution, the R_1_ values (Fig. 3C) can be determined using Eq. 1 from the fast dynamic scan (Fig. 3B) at a high spatial resolution. The baseline R_1_ was subtracted from the time series to calculate the ΔR_1_ maps, which are linearly related to the changes of contrast agent concentration.

### AIF determination

**Figure 4:**
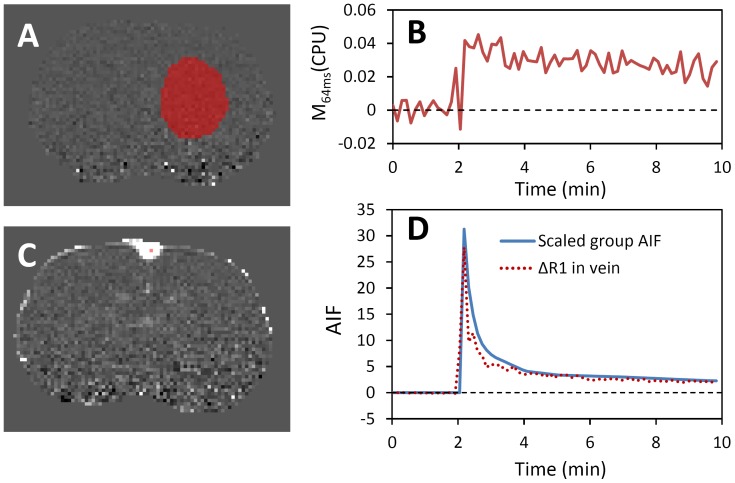
Estimation of AIF. (A) The area selected in contralesional CPU for scaling the group-averaged AIF data. (B) The averaged signal intensity change in CPU from the area determined in (A). (C) The voxel selected to generate the venous input function as shown as the red dotted line in (D). The agreement of the scaled AIF with the determined VIF indicated the validity of the scaling method.

From the ΔR_1_ maps, a region of interest was manually selected in the region of contralesional caudate putamen (Fig. 4A). The signals were averaged to derive the curve shown in Fig. 4B. Using the signal from 3 to 9 min post contrast agent infusion, the group AIF data were scaled assuming a 1% plasma volume in the caudate putamen and that the caudate putamen had no BBB leakage (Fig. 4D, blue solid line). From the raw image, we selected the voxel in the center of superior sagittal sinus to minimize potential partial volume effects (Fig. 4C), which gave a signal profile (Fig. 4D, dotted red line) similar to the scaled group AIF data. This agreement supported the validity of the scaling approach.

### Fitting data to tracer kinetic models

**Figure 5:**
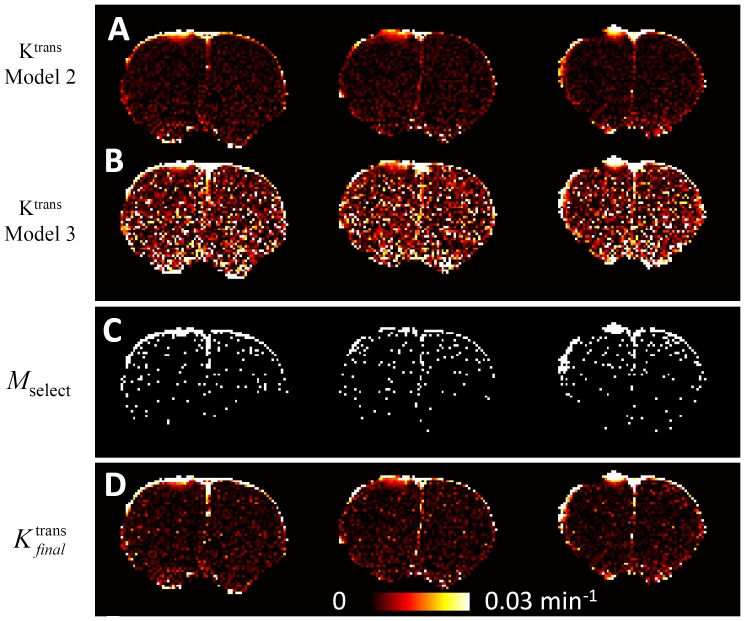
The fitting to different models and model selection. (A) K^trans^ fitted using model 2, the irreversible leakage model. (B) K^trans^ fitted using model 3, the reversible leakage model. (C) Mask for model selection. (D) Final K^trans^ by combining (A) and (B) using the mask as shown in (C) according to Eq. 8.

Using Model 2, the fitted K^trans^ showed good image quality (Fig. 5A). In comparison, the fitted K^trans^ map using Model 3 was significantly noisier (Fig. 5B). Using F-statistics, the majority of voxels could be sufficiently described by Model 2, while much fewer voxels, especially in the injured tissue near the brain boundary, should be fitted by Model 3 (Fig. 5C). The final *K* ^trans^ map (Fig. 5D) was determined by combining the *K* ^trans^ maps obtained using the two models according to Eq. [8]. Using model selection, the final *K* ^trans^ map showed good image quality. The Dice's coefficient between the final *K* ^trans^ and Model 2 is 0.87±0.01, and is 0.13±0.01 between the final *K* ^trans^ and Model 3 (n = 3).

**Figure 6:**
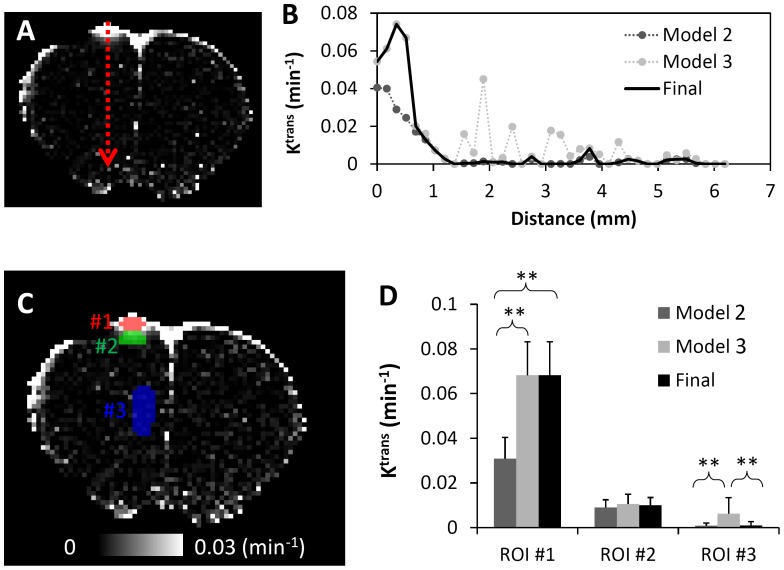
K^trans^ values using different models in a representative TBI brain. A: The location for the selected voxels for the voxel-wise intensity comparison in panel B. C: Label of ROIs. D: The K^trans^ values in these ROIs using different approaches. Data were presented as mean ± standard error of each ROI.

Fig. 6 illustrated the impact of model selection on the K^trans^ determination in this TBI brain. Along the red dotted line (Fig. 6A), Model 3 was selected more in the impact region, and was selected less in the remote region (Fig. 6B), when compared to Model 2. For further comparison, three different ROIs were drawn in the focal lesion, perifocal, and remote regions. The focal lesion region (Fig. 6C, ROI #1) showed the strongest BBB leakage, in which 100% of the voxels were better fit by Model 3 with reversible leakage. If reversible flux was neglected, the K^trans^ value was significantly underestimated (P<0.01, Fig. 6D). In the perifocal region (Fig. 6C, ROI #2), 19% voxels were better fit by Model 3. K^trans^ values determined by Model 2 and Model 3 were similar (P = 0.5). In the healthy region remote from the impact site (Fig. 6C, ROI #3), only 6% of voxels were better fit by Model 3. If reversible leakage was applied to all voxels (Model 3), the K^trans^ was much nosier and significantly overestimated (P<0.01).

### K^trans^ vs. EB extravasation and T_2_

**Figure 7:**
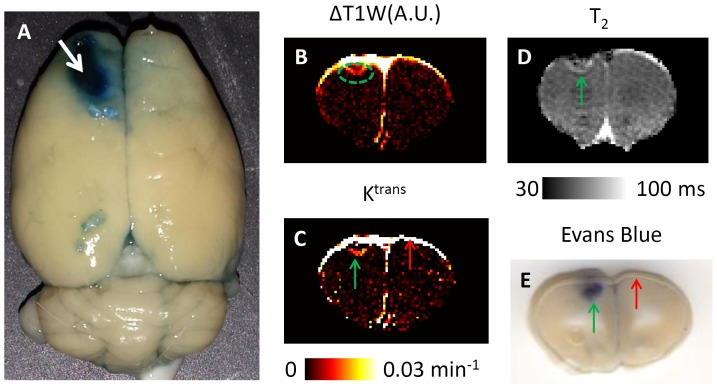
Comparison of EB staining, T_1_-weighted contrast enhancement, T_2_, and K^trans^ map. A: Whole brain images of EB staining. B: T_1_-weighted (T_1_W) signal enhancement. C: K^trans^ map. D: T_2_ map. E: the corresponding axial slice of EB staining. The green arrow pointed to the focal lesion regions showing BBB leakage by both K^trans^ MRI and EB staining. The red arrow shows significant increased intensity in K^trans^ map but no leakage by EB extravasation.

Fig. 7A shows the whole brain with EB staining. Extensive tissue damage can be easily identified as indicated by the white arrow. The T_1_-weighted contrast enhancement (ΔT_1_W, Fig. 7B), K^trans^ map (Fig. 7C) and T_2_ map (Fig. 7D) of the same TBI brain were compared with the corresponding tissue slice with EB extravasation (Fig. 7E). Compared to the qualitative ΔT_1_W with higher SNR, K^trans^ provided slightly lower but reasonably good image quality with the ability to make quantitative measures of BBB permeability. Both K^trans^ and EB staining provided good delineation of BBB leakage in the region of focal lesion (green arrow). Compare to blurred boundaries in EB staining, K^trans^ gave sharper boundaries of BBB leakage. The K^trans^ heterogeneity showed similar features as the T_2_ map. There was a difference in the K^trans^ maps and the EB staining. The K^trans^ maps also showed hyper-intensities at the brain boundaries, whereas no EB staining did not (red arrow).

**Figure 8:**
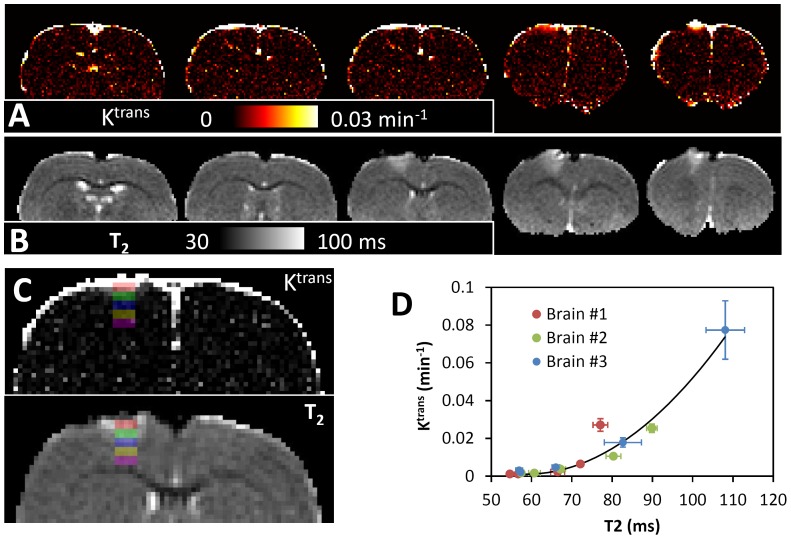
Comparing K^trans^ map with T_2_. **A: K^trans^, B: T_2_, C: ROI labels.** D: K^trans^ and T_2_ values for the same ROIs as shown in C. The solid black line represented the quadratic function (*y* = 0.1037−0.0035*x*+2.98×10^−5^ *x* ^2^) obtained by the fitting.

Fig. 8 compares a K^trans^ map (Fig. 8A) with a T_2_ map (Fig. 8B). Both increased BBB permeability and increased T_2_ were located underneath the impact site. However, the degrees of increase between the two were not the same. A series of ROIs of the impact, the perifocal and the remote regions were drawn in the K^trans^ and T_2_ maps (Fig. 8C). Care was taken to exclude voxels at the edges of the brain (at least 1 voxel) to minimize the effect of hyper-intensity along the boundary of the brain. A nonlinear relationship was observed between the increased T_2_ and the increased BBB leakage (Fig. 8D). The data was fit to a quadratic function (black line). The resulting coefficient for the quadratic term is 3.0×10^−5^, which is statistically significant (P<0.0001).

## Discussion

In this study, we developed a K^trans^ MRI method to quantify BBB leakage with high spatiotemporal resolution for a dynamic scan and sufficient image quality for application in TBI. This method utilized a high field 7 Tesla Bruker BioSpec MRI scanner and a sensitive surface coil. To overcome the signal drop off profile of the surface coil, a 3-scan module was used to determine the flip angle and M_0_ prior to the dynamic scan. The R_1_ maps were then determined using the GRE signal equation with known flip angle and M_0_, and the AIF was determined using scaled group-average AIF data. The obtained K^trans^ map provided a quantitative map of the BBB leakage around the impact region that matches what was outlined by the Evans Blue extravasation. A nonlinear relationship between K^trans^ and T_2_ was observed in the impact site and surrounding regions. This K^trans^ MRI method provided a useful tool for longitudinal monitoring of BBB opening and for investigating the pathophysiology in experimental TBI.

### Impact of surface transceiver coil on R_1_ mapping

Surface coils generally provide better signal sensitivity for preclinical scans than conventional volume coils. However, the use of surface coils for RF transmission leads to significant flip angle variation across the brain. In this study, we used dual-TR FLASH scans to measure the flip angle distribution. Since the T_1_ of the majority of the brain tissue is in the range of 1.4 to 2.1 s, the influence of T_1_ on the flip angle calculation was small (Fig. 1C). As such, a mean value of 1.8 s was used for deriving the raw flip angle map (Fig. 1F). A spatial smoothing was performed since T_1_ variation (in white matter, blood and the CSF) was generally distributed throughout the brain, and the flip angle should not have large local variations. In addition, this flip angle mapping method used the same sequence as the subsequent dynamic scans, thereby avoiding different distortions associated with different sequences.

Another issue with the surface receiver coil was the signal drop off, which was apparent in the long TR scan for M_0_ mapping (Fig. 2). Fortunately, the SNR of this scan is generally high owing to the long scan time. For scans with shorter TRs (for both flip angle mapping and dynamic scans), the signal intensity was much more homogenous (Fig. 1 and Fig. 3), due to the signal increase with smaller flip angles (according to Eq. 1). As a result, the R_1_ values of the deeper structures can be detected without significantly compromising SNR. From Fig. 3A, the sensitivity T_1_-weighted signal to gadolinium concentration was also related to the flip angle (Fig. 3A). With the nominal flip angle of 30^o^, the real flip angle gradually decreases from approximately 45^o^ at the top of the brain to approximately 15^o^ at the bottom of the brain. The T_1_-weighted signal has sufficient sensitivity for this entire range of flip angle distribution.

### Determination of arterial input function

Accurate determination of AIF for preclinical MRI is non-trivial, given the fast arterial blood flow and the small dimensions of rodent brains. As such, different strategies have been proposed to mitigate this difficulty. For example, AIF determined from other imaging modalities has been used for fitting K^trans^ [10], alternative reference region approaches have been developed to eliminate the use of AIF [20], and venous blood signals have also been employed to determine the vascular input function (VIF) instead of AIF [21]. In this study, we adopted the established approach by Ewing and colleagues [14], [15], and used a scaled group average AIF determined previously from radio-labeled Gd-DTPA. Although we could obtain similar VIF profiles from the veins to cross-validate the scaling approach, it should be noted that the accuracy of the VIF is poor due to the wash-in effects and its reproducibility cannot be guaranteed, so we chose to utilize the scaled group AIF data in the model fitting.

### Fitting to tracer kinetic models and model selection

To achieve the desirable image quality for K^trans^, we used model selection as described by Ewing and colleagues [15]. For TBI, the reversible leakage model was necessary for the focal lesion, as shown by (Fig. 6B). Significant underestimation was observed if the reversible leakage was neglected (Fig. 6D). This result indicated that the K^trans^ was sufficiently large, so that the contrast agent can accumulate to a significant amount in the extracellular extravascular space to allow a significant reversible flux of contrast agent back to the plasma. In other region, the irreversible leakage model was sufficient, which is consistent with the existing knowledge that intact brain tissue has negligible contrast agent leakage. Overall, the model selection approach was necessary to ensure that K^trans^ was not underestimated in regions with significant reversible leakage and provided sufficient SNR for the whole brain.

### Spatiotemporal resolution

Look-Locker type of sequences have also been previously used in DCE-MRI studies [22]. While such methods allow independent R_1_ mapping at each time point, it has much lower temporal resolution and lower SNR compared with the gradient-echo methods used in this and other studies [23], [24]. While some recent studies achieved higher temporal resolution (4 to 6 s per image), the 8-s temporal resolution for the dynamic scan in this study was sufficient for TBI studies, since the BBB leakage is generally much lower than in tumors [23], [24]. The slightly lower temporal resolution allows higher spatial resolution and more spatial coverage, which is desirable for TBI studies.

### K^trans^ MRI vs. Evans Blue extravasation

Histological validation of MRI methods is challenging because MRI and histology do not measure exactly the same physiological parameters. K^trans^ MRI indirectly measures the water signals due to the leakage of small-molecular-weight gadodiamide (592Da), whereas the EB method directly measures the leakage of EB bound to serum proteins (65 kDa). The timings for contrast leakage over which the two methods sampled also differ. K^trans^ MRI measures leakage via a single bolus of contrast dynamically, whereas EB measures leakage of accumulated EB over an hour at a steady state. Nevertheless, Fig. 7 shows that the leakage determined using K^trans^ generally matched Evans blue extravasation in the focal lesion region. However, it appeared that EB did penetrate deeper into the surrounding tissue than the gadodiamide contrast agent measured by MRI. Some possible reasons are the differences in molecular weights, timing, interactions with vessel walls and the surrounding cellular components between EB and gadodiamide molecules. Another reason could be further diffusion of EB during the transcardial perfusion with heparinized saline solution. In addition, K^trans^ MRI also showed higher intensities at the brain boundaries. Based on the dynamic signal changes, the increased K^trans^ at the brain boundaries was not from parenchymal tissues or fat from the skull and was not due to partial volume effects. They were likely due to contrast agent leakage from vasculatures outside the brain, or from damaged tissue at the impact site. Therefore, care should be taken when analyzing voxels close to the brain boundary, as the increased K^trans^ value may not represent true BBB leakage. Nevertheless, K^trans^ provides a quantitative measure of BBB leakage non-invasively, allowing the longitudinal measure of BBB changes, something that is not possible using the EB approach. Since EB interferes with lesion staining, additional histological staining was not performed. Previously, a detailed comparison between T_2_ and Nissl staining has been performed [17]. Overall, our results suggest that the K^trans^ heterogeneity follows the lesion variation shown in T_2_ better than EB staining.

### Compatibility with other methods for multi-modal MRI

Our motivation to further develop the K^trans^ MRI method using a surface coil to quantify BBB permeability was to obtain a method that would also be compatible with arterial spin labeling measurements. Arterial spin labeling uses a separate neck coil to measure cerebral blood flow [25], [26], [27]. There is substantial evidence that blood flow is markedly perturbed following TBI but CBF is seldom measured in TBI. Acute and chronic perfusion abnormality could have a negative impact on tissue viability following TBI. Future studies will correlate K^trans^ MRI data with blood flow, diffusion, and T_2_ data longitudinally in the same animal to better understand the pathophysiology of TBI and to better evaluate potential therapies.

## Conclusions

We developed a K^trans^ MRI method for measuring BBB permeability using a surface coil and a gradient-echo based pulse sequence. The obtained K^trans^ map provided excellent SNR, high spatial resolution and spatial coverage. The BBB leakage determined using K^trans^ agrees well with Evans Blue extravasation, and displayed a nonlinear relationship with T_2_. Future studies will use this method to systematically evaluate the longitudinal progression of BBB leakage following TBI. This approach can also be used to investigate other neurological disorders.
